# Psychometric properties of the Fatigue Severity Scale in obese patients

**DOI:** 10.1186/1477-7525-11-32

**Published:** 2013-03-06

**Authors:** Franco M Impellizzeri, Fiorenza Agosti, Alessandra De Col, Alessandro Sartorio

**Affiliations:** 1 Department of Research and Development, Schulthess Clinic, Lengghalde 2, 8008 Zurich, Switzerland; 2 Istituto Auxologico Italiano, IRCCS, Experimental Laboratory for Auxo-endocrinological Research, Piancavallo, VB, Italy; 3 Istituto Auxologico Italiano, IRCCS, Experimental Laboratory for Auxo-endocrinological Research, Milan, Italy; 4 Istituto Auxologico Italiano, IRCCS, Division of Metabolic Diseases and Auxology, Piancavallo, VB, Italy

**Keywords:** Obesity, Symptoms, Questionnaires, Psychometrics

## Abstract

**Background:**

The aim of this study was to examine the psychometric properties of the Fatigue Severity Scale (FSS) to verify whether this instrument is a valid tool to measure fatigue in obese patients, and to examine the prevalence of fatigue in obese patients.

**Methods:**

Before and after a three-week residential multidisciplinary integrated weight reduction program, 220 patients were asked to fill in the questionnaires: FSS, Profile of Mood States (Fatigue-Inertia subscale, POMS-Fatigue, and Vigor-Activity subscale, POMS-Vigor), and the Obesity-Related Well-Being (ORWELL-97). A subsample of 50 patients completed the questionnaire within two days.

**Results:**

The prevalence of fatigue using a cut-off value of 4 for the FSS score was 59%. Correlations were found between FSS and POMS-Fatigue and -Vigor scores (r = 0.58 and 0.53, respectively). A relation was also found between FSS and ORWELL97 (r = 0.52, 0.42 to 0.61). From the factorial analysis only 1 factor was extracted explaining 63% of variance, with factor loading values ranging from 0.71 (item 7) to 0.87 (item 6). Intraclass Correlation Coefficient was 0.89 (0.82 to 0.94), while the agreement as measured using the Standard Error of Measurement was 0.43 (0.36 to 0.54) corresponding to 13% (11 to 17%). Cronbach’s alpha values ranged from 0.94 to 0.93. The internal responsiveness of FSS was comparable to the ORWELL97 (Standardized Response Mean = 0.50 and 0.44, respectively).

**Conclusions:**

Fatigue is an important and frequent symptom in obese patients and therefore should be routinely assessed in both research and clinical practice. This can be achieved using the FSS, which is a short, simple, valid and reliable tool for assessing and quantifying fatigue in obese patients.

## Background

Fatigue is a symptom frequently reported in both healthy [1] and several clinical populations such as patients with depression, rheumatoid disorders, post stroke, multiple sclerosis, mood disorders, psychiatric disturbances and cancer [2]. Given the subjective nature of fatigue, no exact definition exists. From a clinical point of view, fatigue can be defined “as difficulty in initiation or sustaining of voluntary activities” [2] and is characterized by symptoms such as lack of energy, physical and mental tiredness and apathy [2,3].

Fatigue has been associated with obesity in both adult and pediatric populations and is a common complaint in obese people [4-6]. Furthermore, the use of fatigue as a relevant outcome in intervention studies is increasing [7,8]. Since fatigue perception is a subjective experience, it can be measured using self-reports [2,6]. Among the instruments used in obese population there are the Multidimensional Fatigue Symptom Inventory [6], the pediatric Quality of Life Inventory Multidimensional Fatigue Scale [4] and the Fatigue Severity Scale (FSS) [7,8]. The FSS is one of the most commonly used fatigue questionnaires in chronic diseases [9,10] and one of the first instruments applied to obese people [11]. The FSS is a short questionnaire developed by Krupp et al. [12] and consists of 9 items that measure how fatigue affects motivation, exercise, physical functioning, carrying out duties, interfering with work, family, or social life. Given it is a short and simple instrument, it can be implemented in both routine clinical practice and research, and it can be easily added to other patient-reported outcome instruments if additional specific aspects of quality of life would like to be assessed by the researchers or practitioners.

Despite the potential usefulness of the FSS in obese patients and the increased use of this tool for descriptive and intervention studies [7,8], only the study by Grieve et al. [11] has examined the suitability of this questionnaire in obese patients [11]. However, in that study the cohort consisted of only females and few psychometric properties were examined. The aim of this study was to comprehensively examine the measurement properties of the FSS to understand whether the FSS is a valid tool for assessing and quantifying fatigue in obese patients. Specifically we sought to examine: 1) construct validity (i.e. convergent, discriminant and structural validity), 2) internal consistency and reproducibility (reliability, agreement and minimal detectable change), and 3) internal responsiveness in comparison with another specific-questionnaire.

## Methods

### Participants

Two hundred and twenty consecutive obese subjects participated in this prospective (pre-post) observational study. Participants were inpatients referred to the Division of Metabolic Diseases, Istituto Auxologico Italiano, Piancavallo (Verbania, Italy) for a 3-week residential multidisciplinary integrated weight reduction program entailing energy restricted diet, adapted physical activity, psychological and nutritional counseling. Upon admission and after three weeks, patients were asked to fill in the questionnaires. The intervention lasted 3 weeks. As a consequence, while the absolute level of responsiveness was not expected to be large in absolute terms, the comparison with other questionnaires’ responsiveness was still possible and meaningful. To examine absolute and relative reliability a subset of 50 consecutive patients at admission were asked to fill in the questionnaires again after 48 hours. The study was approved by the local Ethics Committee and all patients provided written informed consent to participate.

### Questionnaires

#### Fatigue severity scale

An Italian version of the FSS cross-cultural validated according to available guidelines [13] was used. The FSS consists of 9 statements for evaluating the impact of the fatigue [12]. The subject was asked to rate the severity of the fatigue symptoms experienced in the last week using a numeric scale ranging from 1 (strong disagreement with the statement) to 7 (strong agreement with the statement). The total score has been calculated by averaging the scores of each item.

#### Profile of mood states

The POMS is a questionnaire consisting of 65 items for evaluating six mood states [14] and each item is rated on a 5-point Likert scale (scores from 1 to 5). For the purpose of this study only the Fatigue-Inertia (POMS-Fatigue) and Vigor-Activity (POMS-Vigor) scales were used (for convergent and discriminant validity, respectively). The summary scores of the two scales were calculated according to the developer instructions [14].

#### Obesity-related well-being (ORWELL-97)

The ORWELL 97 consists of 18 items measuring three constructs: symptoms (five items), discomfort (seven items), and impact (six items) [15,16]. For each item the patient has to score, using a 4-point Likert scale, the occurrence and the severity of the symptom (occurrence) and the relevance of the impairment caused by the specific symptom on daily-life (relevance). The score for each item is calculated by multiplying occurrence by relevance score with the higher values indicating a lower condition. Although the single total values for occurrence (ORWELL 97-O) and relevance (ORWELL 97-R) can be calculated in the present study, only the total score was used.

### Statistical analysis

Unless otherwise stated, all data are presented as the mean and standard deviation (SD). Floor and ceiling effects were calculated as the percentage of patients showing, respectively, the lowest and highest values for the instrument. The lowest and highest values were also calculated as the actual minimal and maximal scores for the instruments plus or minus their corresponding Minimal Detectable Change (MDC). This procedure was applied to take into account the error of measurement for the instrument.

#### Convergent and discriminant construct validity

To examine whether the FSS and POMS measured similar (convergent validity with POMS-Fatigue) or dissimilar (discriminant validity with POMS-Vigor) constructs and whether similar constructs changed to the same extent, we calculated the correlations (Pearson’s product moment correlation coefficient) between the absolute values and the change scores after the intervention. We hypothesized a substantial (moderate) positive correlation between FSS and POMS Fatigue, and a negative correlation with POMS Vigor. Correlations were also calculated between FSS and ORWELL-97.

The ability of the FSS to differentiate between patients with a different amount of comorbidities, assuming that those with more comorbidities were also those with more fatigue symptoms, was examined using one-way ANOVA with the factor “number of comorbidities” (5 levels: 0, 1, 2, 3, >3) as independent variable. The correlations between FSS and BMI and age were also calculated using Pearson’s r.

#### Structural validity

Factor structure was examined using exploratory factor analysis, with maximum likelihood factor extraction method and oblique rotation (direct oblimin). The number of components were determined using the scree-test on the sedimentation graph and the Kaiser’ s criterion, which requires eigenvalues > 1 [17].

#### Reproducibility and internal consistency

Reliability was calculated using the intra-class correlation coefficient (two-way mixed, single measure model) while agreement was determined by calculating the Standard Error of Measurement (SEM) [18]. The 95% Confidence Interval for ICC and SEM was also reported. The minimal detectable change (MDC) at the individual level was calculated as SEM x √2 x 1.96. Systematic bias was examined using a paired *t*-test. Data are presented in absolute and relative (percentage) terms with percentages calculated after log transformation. Internal consistency was calculated using Cronbach’s alpha coefficient (CA).

#### Internal responsiveness

We calculated internal responsiveness using the Cohen’s d effect size [ES = (posttest mean – pretest mean)/SD baseline] and standardized response mean [SRM = (posttest mean – pretest mean)/SD changes] [18-22]. The confidence intervals (95%) for ES and SRM were also calculated. Changes in the dependent variables were analyzed using a paired *t*-test and reporting the mean difference with the corresponding 95% confidence intervals.

The strength of the correlations were interpreted using the Cohen’s benchmarks: <0.10, trivial; 0.10 to 0.30, small; 0.30 to 0.50, moderate; >0.50, large [23]. P values <0.05 were considered to be statistically significant under the null-hypothesis paradigm. The analyses were conducted using SPSS (version 17 SPSS Inc, Chicago, IL, USA)

## Results

### Descriptive data

The baseline socio-demographic characteristics of the patients participating in the study are shown in Table 1. The prevalence of fatigue using a cut-off value of 4 [3] for the FSS score was 59%.

**Table 1:** Baseline socio-demographic characteristics of the patients participating in the study (n = 220).

**Variables**	**Unit**	**Whole group**	**Sub-sample** ** for reproducibility**
Female	N (% of total)	154 (70%)	36 (72%)
Male	N (% of total)	66 (30%)	14 (28%)
Age	Years (SD)	47 (15)	47 (12)
BMI	kg/m^2^	44.4 (5.2)	44.5 (5.8)
Education	Primary school	51%	50%
	High school	39%	38%
	University	10%	12%
Occupation	White collars	22%	24%
	Blue collars	24%	28%
	Retired	24%	24%
	Students	5%	4%
	Homeworkers	15%	10%
	Not employed	9%	10%
	Other	1%	-

There were no missing data given the questionnaires were filled under the control of operators. Only 3 patients reported at baseline the lowest FSS scale value (floor effect) and 3 the highest value (ceiling effect). Taking into account the MDC, the floor and ceiling effect at baseline was 10%. Post-intervention, 7 subjects reported the lowest value and only 1 the highest. Taking into account the MDC, the floor and ceiling effect post-intervention was 5%. The floor and ceiling effects were lower than the 15% cut-off value considered acceptable [24,25].

#### Evidence of construct validity

At baseline positive and large correlations were found between FSS and POMS Fatigue score (r = 0.58; 95% CI 0.48 to 0.66). Similarly, the correlation between FSS and POMS Vigor was significantly negative and moderate-large (r = −0.53; 0.43 to 0.62). Similar figures were found using the data collected post intervention (results not shown). The correlation between the change scores in FSS and POMS-Fatigue was positive and moderate (r = 0.41, 0.29 to 0.51), while with POMS-Vigor the correlation was negative and small (r = −0.26, 0.13 to 0.38). Moderate-large correlations were also found between FSS and ORWELL97 (r = 0.52, 0.42 to 0.61), while a small-moderate correlation was found between change scores of the two questionnaires (r = 0.29, 0.16 to 0.41).

**Figure 1:**
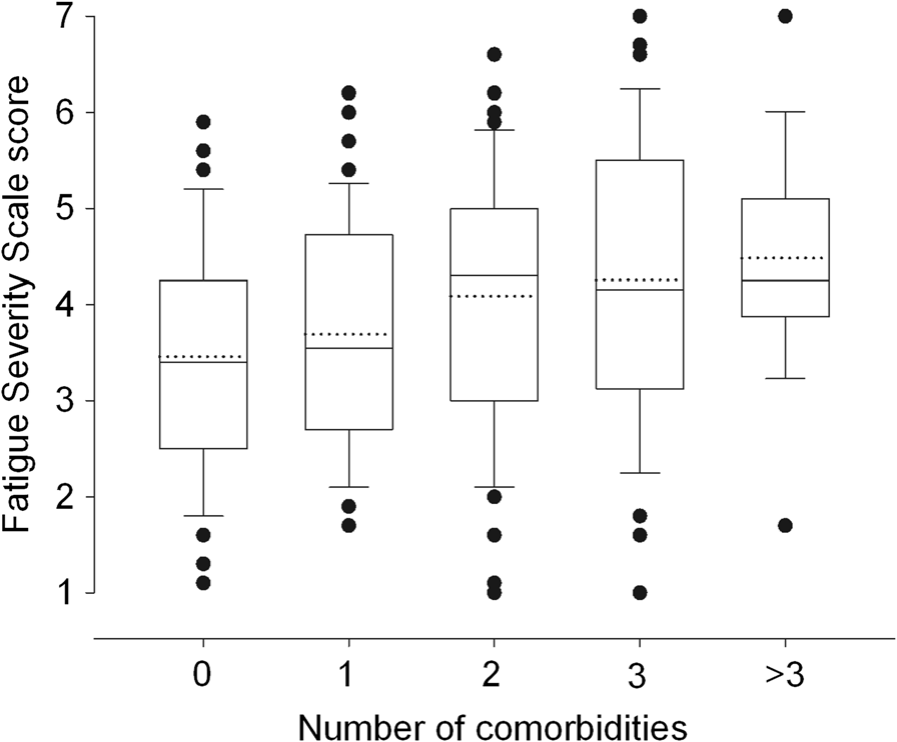
**Boxplots showing median (horizontal black line), mean (dotted black line), interquartile range (box), error bar (10**^**th **^**and 90**^**th **^**percentiles), and outliers (black circles) of the FSS in obese patients classified according to the number of comorbidities.**

Differences in FSS values (p < 0.001) were found for groups of patients categorized according to the number of comorbidities (Figure 1). A significant correlation was found between age and FSS (r = 0.28, 0.15 to 0.40). The correlation between FSS and BMI was significant but small (r = 0.15, 0.02 to 0.28). However, when adjusted for age the correlation increased to r = 0.25 (0.12 to 0.37).

From the factorial analysis only 1 factor was extracted explaining 63% of variance, with factor loading values ranging from 0.71 (item 7) to 0.87 (item 6).

#### Reproducibility and internal consistency

No substantial systematic bias (mean difference: -0.2, 95% CI 0.0 to −0.4) was found between test [4.0 (SD 1.3)] and retest [3.8 (SD 1.3)] FSS values. Reliability as measured using the ICC was 0.89 (0.82 to 0.94), while the agreement as measured using the SEM was 0.43 (0.36 to 0.54) corresponding to 13% (11 to 17%). The MDC was 1.2 points (37%). Cronbach’s alpha was 0.94 and 0.93 at baseline and post-intervention, respectively (Table 2).

**Table 2:** Internal consistency (Cronbach’s Alpha) of the Fatigue Severity Scale

	**Baseline**		**After intervention**	
**Item**	**Corrected item-total correlation**	**Cronbach's alpha if item deleted**	**Corrected item-total correlation**	**Cronbach's alpha if item deleted**
1	0.73	0.93	0.70	0.92
2	0.72	0.93	0.41	0.95
3	0.79	0.93	0.80	0.91
4	0.77	0.93	0.85	0.91
5	0.81	0.93	0.83	0.91
6	0.84	0.93	0.86	0.91
7	0.69	0.94	0.75	0.92
8	0.83	0.93	0.84	0.91
9	0.75	0.93	0.76	0.92

#### Internal responsiveness

Significant moderate changes were found for all the instruments and BMI after the three-week intervention (Table 3). The internal responsiveness of FSS was comparable to the ORWELL97.

**Table 3:** Internal responsiveness and pre-post changes of the various patient-reported outcomes

	**Pre**	**Post**	**Pre-post difference**	**ES ( *d *)**	**SRM**
	**Mean (SD)**	**Mean (SD)**	**Value (95% CI)**	**Value (95%CI)**	**Value (95%CI)**
**BMI**	44.2 (5.2)	42.7 (6.0)	−1.6 (−1.1 to −2.0)	−0.31 (−0.03 to −0.58)	−0.53 (−0.25 to −0.80)
**ORWELL-97**	51.5 (27.9)	43.0 (27.0)	−8.4 (−5.7 to −11.1)	0.30 (−0.03 to −0.57)	−0.44 (−0.17 to −0.72)
**FSS Total score**	3.9 (1.3)	3.4 (1.3)	−0.5 (−0.4 to −0.7)	−0.37 (−0.10 to −0.65)	−0.50 (−0.22 to −0.78)
**POMS-Fatigue**	9.2 (5.7)	6.5 (5.4)	−2.7 (−2.0 to −3.3)	−0.47 (−0.20 to −0.75)	−0.55 (−0.27 to −0.83)
**POMS-Vigor**	14.9 (6.9)	17.3 (6.9)	2.3 (1.6 to 3.1)	0.33 (0.06 to 0.61)	0.42 (0.14 to 0.69)

## Discussion

This study showed that the FSS is a valid instrument for measuring fatigue in obese patients. The results of the current investigation provided good evidence of validity and reliability, suggesting this patient-reported outcome tool is suitable for both cross-sectional and longitudinal assessments of fatigue in both practice and research settings.

### Construct validity

To build up a body of evidence to support the validity of a measure, several attributes should be examined [26,27]. In the present study, we examined the convergent validity of the FSS with the POMS-Fatigue score as it is supposed to measure the same construct. The correlation was significant and close or higher than the 0.50 cut-off correlation value considered the minimum for showing adequate construct validity. However, the lower limit of the confidence intervals was below this cut-off value and a correlation of 0.50 means that only 25% of the variance is shared. Therefore, although the two instruments measured a similar construct, they probably assessed different aspects of fatigue. Indeed, while the POMS-Fatigue scale has been suggested to be a measure of the fatigue severity, the FSS is considered a measure of both severity and impact of fatigue on daily life [10].

We also examined the discriminant validity (ability to discriminate dissimilar constructs) by assessing the relation between FSS and POMS-Vigor score, which we hypothesized, would go in the opposite direction. According to our theory, the relation between FSS and POMS-Vigor was negative. Moderate correlations were also found between the change scores of the FSS and POMS scales further confirming the two instruments measured similar constructs. The correlations between change scores can also be interpreted as evidence (moderate) of external responsiveness that has been defined as the ability of a questionnaire to detect change over time in the construct to be measured [10,24]. However, since there are no reference measures for assessing fatigue in obese population, we prefer to consider this finding a further evidence of convergent construct validity. As further evidence of construct validity we compared the FSS scores of obese patients concurrently experiencing other comorbidities, therefore assuming that the more the comorbidities the higher the level of fatigue. As hypothesized, we found that the level of fatigue increased with the number of comorbidities. Lastly, the factor analysis confirmed the 1-factor structure of the FSS, explaining 63% of the variance, which is a value higher than the 0.50 cut-off considered necessary for confirming the structure validity [10,24]. The amount of variance explained found in the present study is similar to the 67.7% recently reported by Ferentinos et al. in patients with major depression [28]. Overall, the aforementioned findings support the construct validity of the FSS as measure of fatigue in obese patients.

### Reproducibility and internal consistency

When exploring the reproducibility of an instrument both reliability and agreement should be determined [18]. The reliability of the FSS as measured using the ICC was 0.89, which is usually considered a high value and thus suggesting the FSS is potentially an adequate instrument for cross-sectional comparisons. The agreement (measurement error) as measured using the SEM was 0.4 corresponding to 13% of the mean FSS values. Based on this SEM, the MDC (with 95%CI) was 1.2 points (37%) meaning that individual changes higher than these figures can be interpreted as real and not due to measurement error with an acceptable probability level. The SEM and MDC reported can be used for helping to appropriately interpret the changes in the practical setting. Overall, the reproducibility was found to be good and adequate to use the FSS for both differentiating between individuals and for longitudinal assessments.

Similar to previous studies, the FSS showed excellent internal consistency [3,10]. Valko et al. [3] reported in patients with various disorders usually associated to fatigue, the lowest internal consistency for items 1 and 2. Our analysis showed the lowest values for items 1,2 and 7 both before and after the three-week intervention. Therefore, our results are partly in agreement with Valko et al. [3]. In a series of recent studies examining the psychometric properties of the FSS using Item Response Theory in stroke, multiple sclerosis and HIV-infected adults, high mean square values were found for items 1 and 2, and a reduced version (FFS-7) has been proposed [29-31]. The results of these previous studies and our findings seems to suggest that further studies using classical and/or modern test theory are probably needed to understand whether a reduced version may show even better measurement properties for use in obese patients.

### Internal responsiveness

After the three-week intervention the scores of the patient-reported outcomes improved significantly. The responsiveness as measured using ES ranged from 0.30 to 0.47 and from 0.42 to 0.55 for SRM. Overall, these figures indicated that the entity of improvements after the intervention was moderate which was not surprisingly given the short duration of the intervention that aimed to provide the foundations for future changes. However, the main purpose was to compare the internal responsiveness of the FSS with the other instruments and particularly with disease-specific questionnaires that are usually responsive. The results showed that the ES and SRM values of the FSS were comparable with those found for the ORWELL97, which is a specific instrument developed for assessing obesity-related quality of life taking into account both the intensity and the subjective relevance of physical and psychosocial distress [15,16]. A significant correlation was also found between absolute and change scores of the FSS and ORWELL97. Since ORWELL97 measures a different construct than fatigue, we interpreted the significant correlation as the confirmation of the association between fatigue perception and health-related quality of life of obese patients. Therefore, the FSS may be a complementary tool to other specific instruments that are used to evaluate other aspects of health-related quality of life.

### Fatigue in obese patients

The prevalence of fatigue in this cohort of 220 patients using 4 as cut-off value [3] was 59%. This figure is comparable to what is reported in Parkinson patients (58%), stroke patients (49%), and patients with sleep-wake disorders (62%) [3,32]. Although in other clinical populations the prevalence of fatigue is higher (e.g. multiple sclerosis [3,33]), the figure found in the current study confirms that fatigue is a relevant symptom in this population and as a consequence should be assessed more frequently. Although fatigue is a common symptom in several diseases and chronic conditions including obesity, it is quite surprising that fatigue was not included as a separate category in the International Classification of Functioning, Disability and Health (ICF) [34] as also remarked by Newman [35]. However, the dimension/trait “fatigue” is addressed by the ICF category b130 (“energy and drive functions”) [36], which is one of the 9 categories selected and included by a panel of international experts in the Brief ICF core set for obesity [37]. This further confirms the clinical importance of assessing fatigue in obese patients. Lastly, fatigue not only influences the quality of life but may be an additional factor contributing to the exacerbation of the obese condition. As suggested for cancer patients (for which fatigue is a severe symptom) and more recently for obese too [6,38], fatigue may reduce the level of physical activity making it more difficult to counteract the fatigue itself and the weight management. Therefore, fatigue can create a vicious cycle and a self-perpetuating condition. This area certainly necessitates future studies.

### Limitations

Although the approach used in this study for examining the psychometric properties of the FSS is traditional and well established, other methods such as Item Response Theory are warranted for further confirming the validity of the FSS in obese patients. Furthermore, the FSS measurement properties found in the present cohort cannot be automatically extended to obese patients undergoing other treatments such as bariatric surgery, especially in relation to the sensitivity to changes. While promising, other studies are necessary to provide more evidence of validity of the FSS in obese population and for better interpreting the results (e.g. minimal clinically important changes).

## Conclusion

In conclusion, fatigue is an important and frequent symptom in obese patients affecting quality of life, which may also influence weight management. The FSS is a short, simple, valid and reliable tool for assessing and quantifying fatigue in obese patients. For these reasons, the FSS can and should be used more frequently in both clinical practice and research. Further studies examining the impact of fatigue on obesity and how to manage this symptom for its potential role as a mediator in obesity treatments are warranted.

## Competing interest

The authors declared that they have no competing interest.

## Authors’ contribution

FMI managed the study from conception to publication (conception, design, analysis, interpretation); AS and FA participated in the conception of the study, patient enrollment, data collection, and the revision of the manuscript. ADC collected the data and collaborated in the data analysis. All author read and approved the final manuscript.
